# Glomangiosarcoma arising from traumatic lesion in the elbow^☆^

**DOI:** 10.1016/j.abd.2024.05.010

**Published:** 2025-01-10

**Authors:** Nelson Lobos, María José Pereira, Macarena Stevenson, Dan Hartmann, Valentina Darlic, Alex Castro

**Affiliations:** a Dermato-Oncology Department, Instituto Nacional del Cáncer, Santiago, Chile; b Dermatology Department, Faculty of Medicine, Universidad de Chile, Santiago, Chile; c Dermatology Department, Clinica Alemana, Universidad del Desarrollo, Santiago, Chile; d Faculty of Medicine, Universidad Finis Terrae, Santiago, Chile; e Faculty of Medicine, Universidad del Desarrollo, Santiago, Chile; f Pathology Department, Clinica Alemana, Universidad del Desarrollo, Santiago, Chile

*Dear Editor,*

Glomus Tumors (GTs) originate from the glomus body, an arteriovenous anastomosis located in the reticular dermis,^1^ and are categorized into benign, uncertain behavior, and Malignant Forms (MGTs) based on clinicopathological features.^2^ MGTs, constituting less than 1% of cases, exhibit aggressive local invasion, a tendency for recurrence after excision, and occasional distant metastasis.^1^, ^3^ MGTs are further subclassified into locally infiltrative GTs, Glomangiosarcoma (GS) arising from benign glomus tumors, and de novo GS.^1^, ^4^, ^5^

We present the case of a female patient, 53 years old, with a medical history of knee osteoarthritis, glucose intolerance, obesity, asthma, and hypothyroidism. The patient reported a sharp accident with glass on the outside of the right forearm, evolving with intense neuropathic pain during the first year. Two years later, she reported increased volume in this area, very sensitive to palpation and associated with spontaneous hemorrhage. The physical examination showed a grayish-brown 10 × 6 cm well-demarcated, firm, tender, and pulsatile multilobed tumor with a violaceous hue, on the extensor surface of the right forearm (Fig. 1A‒B). Doppler study showed a mass characterized by a high arterial density that invaded the cutaneous and subcutaneous tissue up to the superficial fascia. An incisional biopsy revealed a glomangiosarcoma. PET scan reveals no distant dissemination. Tumor was excised with 3 cm margins up to the depth of the underlying muscular fascia and with axillary lymph node dissection. Histopathology showed a well-circumscribed dermal and hypodermal tumor composed of cells with eosinophilic cytoplasm, pleomorphic nuclei, prominent nucleoli, atypical mitotic figures, and high mitotic rate (9 mitotic figures/10 HPF) (Fig. 2A-B). Immunohistochemistry was positive for smooth muscle actin (Fig. 2C) and negative for CD31, CD34, S100, and cytokeratin. Ki67 was positive in 20% of tumor cells.

**Fig. 1:**
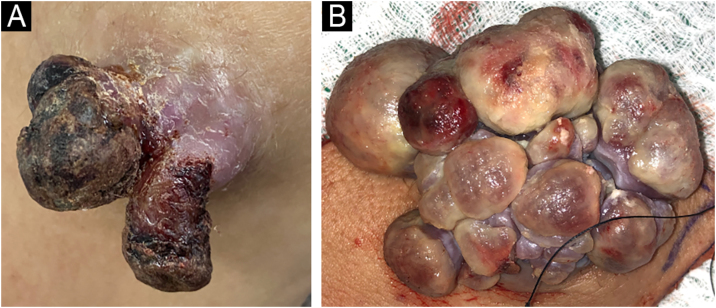
(A) Clinical image of primary exophytic tumor located on the left elbow, with a hemorrhagic crust on the surface at one month of evolution. (B) Clinical image showing a multilobed appearance of the primary tumor after 4 months of rapid growth.

**Fig. 2:**
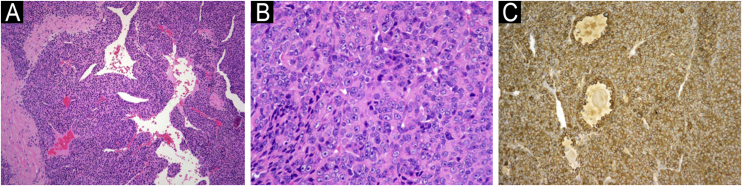
(A) At low magnification, neoplasm consists of round cells around vascular spaces (Hematoxylin & eosin, ×40). (B) Higher magnification shows atypical glomus cells with pleomorphic nuclei and prominent nucleoli. Numerous mitotic figures are seen (Hematoxylin & eosin, ×200). (C) Tumor cells are positive for actin (Anti-Smooth Muscle Actin, ×100).

Most GTs are benign neoplasms that develop in the dermis or subcutaneous tissue of distal regions of the extremities,^5^ where 80% are located in the upper extremities, being especially common in the subungual region.^6^ The majority present as painful, solitary, slow-growing nodules.^1^ MGTs are a rare occurrence, characterized histologically by nuclear atypia, necrosis, and high mitotic index.^1^, ^7^ Gould et al. subclassified MGTs into three categories based on their characteristics in histological studies;^8^ locally infiltrative GTs, which are histologically identical to GT except for their infiltrative growth; GS arising from benign glomus tumors, which are tumors that arise from preexisting GT and contain nuclear pleomorphism and mitotic figures; and GS arising de novo, which are tumors with a high mitotic rate but lack histologic findings of a benign glomus tumor. Most GS arises from a preexisting glomus tumor, across a wide age range of 20‒80 years, without sex predilection, predominantly in the upper and lower limbs.^1^, ^2^, ^8^ Histologically, GS is composed of uniform, round, or oval cells, with eosinophilic cytoplasm, numerous vascular spaces, and cellular pleomorphism associated with frequent mitotic figures, characteristics not present in benign GT.^1^ In immunohistochemical studies, malignant glomus cells stain positively for vimentin, smooth muscle actin, and muscle-specific actin.^1^, ^3^, ^7^ In contrast, these tumors are negative for markers, such as desmin, cytokeratin, CD34, and S100 protein.^7^ MGTs exhibit locally invasive behavior, a tendency for recurrence after excision, and usually do not metastasize.^1^, ^5^, ^8^ Metastatic potential correlates with deep location, size exceeding 2 cm, presence of atypical mitotic figures, or a combination of moderate to high nuclear grade and mitotic activity.^1^, ^2^, ^3^ Wide local excision with negative surgical margins is the primary treatment. Since metastasis is rare, sparse literature exists about the treatment of recurrent and metastatic disease.^1^

In Conclusion, GS carries metastatic risks, particularly associated with deep-seated tumors, substantial size, and the presence of atypical mitotic figures. Wide surgical excision is crucial to prevent recurrence.

## Authors’ contributions

Nelson Lobos: Approval of the final version of the manuscript; critical literature review; manuscript critical review; preparation and writing of the manuscript.

María José Pereira: Critical literature review; manuscript critical review; preparation and writing of the manuscript.

Macarena Stevenson: Critical literature review; manuscript critical review; preparation and writing of the manuscript.

Dan Hartmann: Critical literature review; manuscript critical review; preparation and writing of the manuscript.

Valentina Darlic: Critical literature review; manuscript critical review; preparation and writing of the manuscript.

Alex Castro: Critical literature review; manuscript critical review; preparation and writing of the manuscript.

## Financial support

None declared.

## Conflicts of interest

None declared.
